# Hemoglobin concentrations and RBC transfusion thresholds in patients with acute brain injury: an international survey

**DOI:** 10.1186/s13054-017-1748-4

**Published:** 2017-06-17

**Authors:** Rafael Badenes, Mauro Oddo, José I. Suarez, Massimo Antonelli, Jeffrey Lipman, Giuseppe Citerio, Fabio Silvio Taccone

**Affiliations:** 1 Department of Anesthesiology and Surgical Intensive Care, Hospital Clinic Universitari, Valencia, Spain; 2 Department of Intensive Care Medicine, Centre Hospitalier Universitaire Vaudois (CHUV), Lausanne University Hospital, 1011 Lausanne, Switzerland; 3 Division of Vascular Neurology and Neurocritical Care, Department of Neurology, Baylor College of Medicine, Catholic Health Initiatives (CHI) Baylor St. Luke’s–Baylor St. Luke’s Medical Center, Houston, TX USA; 4 Department of Anesthesiology and Intensive Care Medicine, Catholic University – Fondazione Policlinico Agostino Gemelli University Hospital, Rome, Italy; 5 Intensive Care Services, Royal Brisbane and Women’s Hospital, Herston, Australia; 6 Burns Trauma Critical Care Research Centre, University of Queensland, Herston, Australia; 7 School of Medicine and Surgery, University of Milano-Bicocca, Monza, Italy; 8 Neurointensive Care, San Gerardo Hospital, Azienda Socio Sanitaria Territoriale (ASST) of Monza, Monza, Italy; 9 Department of Intensive Care, Erasme Hospital, Free University of Brussels (ULB), Route de Lennik, 808-1070 Brussels, Belgium

**Keywords:** Hemoglobin, Transfusion, Threshold, Brain injury, Outcome

## Abstract

**Background:**

The optimal hemoglobin (Hb) threshold at which to initiate red blood cell (RBC) transfusion in patients with acute brain injury is unknown. The aim of this survey was to investigate RBC transfusion practices used with these patients.

**Methods:**

We conducted a web-based survey within various societies of critical care medicine for intensive care unit (ICU) physicians who currently manage patients with primary acute brain injury.

**Results:**

A total of 868 responses were obtained from around the world, half of which (*n* = 485) were from European centers; 204 (24%) respondents had a specific certificate in neurocritical care, and most were specialists in anesthesiology or intensive care and had less than 15 years of practice experience. Four hundred sixty-six respondents (54%) said they used an Hb threshold of 7–8 g/dl to initiate RBC transfusion after acute brain injury, although half of these respondents used a different threshold (closer to 9 g/dl) in patients with traumatic brain injury, subarachnoid hemorrhage, or ischemic stroke. Systemic and cerebral factors were reported as influencing the need for higher Hb thresholds. Most respondents agreed that a randomized clinical trial was needed to compare two different Hb thresholds for RBC transfusion, particularly in patients with traumatic brain injury, subarachnoid hemorrhage, and ischemic stroke.

**Conclusions:**

The Hb threshold used for RBC transfusion after acute brain injury was less than 8 g/dl in half of the ICU clinicians who responded to our survey. However, more than 50% of these physicians used higher Hb thresholds in certain conditions.

**Electronic supplementary material:**

The online version of this article (doi:10.1186/s13054-017-1748-4) contains supplementary material, which is available to authorized users.

## Background

Anemia is highly prevalent during critical illness; 60% of patients admitted to intensive care units (ICUs) are considered anemic, and 20**–**30% have a first hemoglobin (Hb) concentration less than 9 g/dl [1]. Moreover, after a 1-week ICU stay, up to 80% of patients will have Hb concentrations below this value [2]. Cohort studies have suggested a strong association between anemia and poor outcome in heterogeneous populations of critically ill patients [3, 4], especially among those with a history of cardiovascular disease [5]. However, the optimal threshold to trigger red blood cell (RBC) transfusion in ICU patients is not clearly established and may depend on other confounders related to the underlying disease or to patient characteristics.

It has been shown that tissue oxygen delivery (DO_2_) is dependent on organ blood flow and arterial oxygen content, which includes the Hb concentration and its oxygen saturation [6]. Thus, during anemia, an increase in local blood flow or in oxygen extraction is necessary to maintain adequate DO_2_ and avoid tissue hypoxia. In healthy conditions, brain tissue has a high oxygen extraction rate, which may limit its ability to further compensate for reduced DO_2_ [7]. Thus, cerebral vasodilation and increased cerebral blood flow (CBF) are the main adaptive responses observed in the brain during anemia. In healthy volunteers, acute isovolemic anemia with Hb concentrations around 5 g/dl was associated with cognitive deficits and impaired central processing, suggesting that maximal vasodilation had been achieved at these Hb concentrations and that no further increase in CBF could be obtained to compensate for reduced DO_2_ [8]. However, in acute brain injury, cerebral oxygen consumption may already be increased and cerebral vasodilation may be severely impaired, so that brain hypoxia could occur at higher Hb concentrations.

Several clinical studies have suggested that Hb concentrations less than 9 g/dl are associated with tissue hypoxia, metabolic crises, and poor outcomes among patients with traumatic brain injury (TBI), subarachnoid hemorrhage (SAH), or intracranial hemorrhage (ICH) [9–11]. However, in the same setting, RBC transfusion has been associated with poor neurological recovery [12, 13]. Thus, assessment of the risk/benefit ratio for transfusion is a key consideration in critically ill patients with acute brain injury [14]. Transfusion practice has moved toward more restrictive strategies in critically ill patients (i.e., to keep Hb ≥7 g/dl) in recent years; however, more liberal targets (e.g., Hb ≥9 g/dl) are recommended in patients with underlying cardiovascular disease [15]. Although this approach may also be logical in patients with acute brain injury, as was recommended in recent guidelines (i.e., to keep Hb between 8 and 10 g/dl) [2, 16], patients with acute brain injury were poorly represented in large clinical trials of RBC transfusion strategies, and further clinical investigation is needed in these patients.

To better understand the feasibility of such studies and how to develop future therapeutic protocols, it is important to assess transfusion practice of ICU physicians who manage patients with acute brain injury. Two surveys of transfusion practice in patients with TBI and SAH have been published [17, 18], but they were limited to cohorts of physicians in North America. Thus, the aim of this survey was to investigate, at a more global level, the management of anemia and RBC transfusion practice, including the Hb threshold for transfusion, in patients with acute brain injury.

## Methods

This study was approved and endorsed by the European Society of Intensive Care Medicine (ESICM) Research Committee. No ethical approval was needed for the participating physicians.

### Study design and administration

We conducted a web-based survey sent by email to all members of the ESICM, the Neurocritical Care Society, the Australian and New Zealand Intensive Care Society, the Società Italiana di Anestesia Analgesia Rianimazione e Terapia Intensiva, and the Brazilian Society of Critical Care (the questionnaire was not translated). The first email was sent on 21 May 2013, with two reminders sent 2 and 4 weeks thereafter, respectively. We did not specifically limit the survey to physicians working in academic institutions; our target sampling was intensivists who regularly care for patients with acute brain injury (i.e., neurointensivists, vascular neurosurgeons, neurologists) or with extensive interest and experience in the management of patients with acute brain injury. The survey was self-administered by the respondents, voluntary, and submitted online using a SurveyMonkey system (SurveyMonkey, San Mateo, CA, USA). No reimbursement was offered for questionnaire completion. All responses were anonymous.

### Survey development

The survey was developed by two investigators (FST, MO) on the basis of a review of the relevant literature in this field. The questionnaire was presented at the Neuro-Intensive Care Section of the ESICM meeting in October 2012 and sent by email to all participants of this section to obtain further input. A consensus was then achieved, and the final version of the survey was sent to the ESICM Research Committee, which gave its final endorsement after a peer review process (i.e., two reviewers).

The survey was constructed using 24 multiple-choice questions (Additional file 1: Appendix 1) to evaluate physicians’ preferences for correcting anemia in patients with acute brain injury. We also recorded details of ICU and physician characteristics (i.e., country, specialty, position, years since completion of training, type of ICU, primary and secondary specialties, number of ICU beds, hospital size, and characteristics). We asked the respondents which Hb threshold they used to trigger RBC transfusion in patients with acute brain injury and whether the type of disease, patient characteristics, or specific conditions (e.g., increased intracranial pressure or vasospasm) would modify these thresholds. We also asked about the diagnosis and management of low iron levels and about the administration of erythropoietin. Finally, we asked clinicians about the potential risks and benefits of RBC transfusion in this setting and whether they believed a clinical trial should be conducted to specifically determine the optimal Hb threshold in this patient population. The survey was not specifically tested in a pilot cohort of potential respondents but underwent a peer review process within the ESICM Research Committee.

### Definitions

We planned a priori to determine how transfusion practices might be influenced by geographical factors and categorized five world areas (Europe, North America, Central and South America, Asia/Africa, and Oceania). We also categorized Hb thresholds as a binary variable: less than 9 g/dl or greater than or equal to 9 g/dl. We defined the factors that could influence RBC transfusion policy as noncerebral (e.g., active bleeding, coronary artery disease[CAD], low mixed venous oxygen saturation [SvO_2_], age, lactate level greater than 2.5 mEq/L) or cerebral (e.g., low brain tissue pressure levels [PbtO_2_ less than 15 mmHg], delayed cerebral ischemia [DCI], data from other neuromonitoring tools [including electroencephalography, noninvasive cerebral oxygenation, transcranial Doppler], presence of intracranial hypertension, Glasgow Coma Scale score less than 9). Types of acute brain injury were separated as follows: TBI, SAH, ischemic stroke, ICH, postneurosurgery, seizure and status epilepticus (SE), hypoxic-ischemic encephalopathy (HIE), central nervous system (CNS) infections, and noninfectious and autoimmune encephalitis.

### Statistical analysis

Discrete variables are expressed as count (percent) and continuous variables as median (IQR or range, as indicated). Differences between study groups were assessed using a chi-square test, Fisher’s exact test, Kruskal-Wallis test, or Mann-Whitney *U* test as appropriate. We performed multivariable logistic regression analyses to identify whether the following issues could be explained by the respondent’s primary specialty (internal medicine, intensive care, anesthesiology, neurology, surgery, or pediatrics), the period since completion of training (years of practice), the type of ICU (medical, neuro-ICU, surgical), or institution (university, university-affiliated, non-university-affiliated) rather than the geographical difference itself: (a) the Hb threshold used to initiate RBC transfusion after acute brain injury, (b) use of noncerebral vs. cerebral factors to adjust the Hb threshold for transfusion, and (c) the concentration of Hb used to initiate RBC transfusion in the presence of one or more influencing factors. The collinearity between variables was checked by inspection of the correlation between them, by looking at the correlation matrix of the estimated parameters, and by looking at the change of parametric estimates and at their estimated standard errors. No instability of parametric estimates or an excessive increase of standard errors was observed during the analyses. A Hosmer-Lemeshow goodness-of-fit test was considered to assess model calibration (agreement between observed outcomes and predictions). Q–Q plots were drawn to check for normality in the residuals. Results are given as ORs and their 95% CIs. Statistical analyses were performed using the IBM SPSS Statistics 24.0 for Windows NT software package (IBM, Armonk, NY, USA). All reported *p* values are two-sided. A *p* value less than 0⋅05 was considered to indicate statistical significance.

## Results

### Survey response and demographic characteristics

A total of 868 completed surveys were obtained from the five regions, 55% of which (*n* = 485) were from European ICUs (Additional file 1: Appendix 2 and Additional file 2: Figure S1). The countries with more respondents were Italy (*n* = 133), the United States (*n* = 129), the United Kingdom (*n* = 69), Switzerland (*n* = 58), and Australia (*n* = 57). Almost one-fourth of respondents (*n* = 204 [24%]) had a specific certificate in neurocritical care (Table 1), with the highest proportion observed in North America. Most respondents (*n* = 729 [84%]) were specialists in anesthesiology or critical care (Table 1); a high proportion of respondents from North America said their primary specialty was neurology. Overall, only 20% of the respondents worked in a specialized neuro-ICU, but this proportion was significantly higher in North America than in other areas. Respondents from North America reported larger numbers of available ICU beds, more total patients, and more patients with acute brain injury admitted to the ICU on the day of the survey.

**Table 1:** Characteristics of respondents and their intensive care units

	Overall (*n* = 868)	Europe (*n* = 485)	North America (*n* = 140)	Central and South America (*n* = 87)	Asia/Africa (*n* = 88)	Oceania (*n* = 68)	*p* Value
Certificate							<0.001
Neurocritical care, *n* (%)	77 (9)	11 (3)	59 (42)	4 (5)	3 (3)	–	
Critical care, *n* (%)	532 (61)	321 (66)	32 (23)	63 (72)	61 (69)	55 (81)	
Both, *n* (%)	127 (15)	75 (15)	31 (22)	12 (14)	5 (6)	4 (5)	
None, *n* (%)	132 (15)	78 (16)	18 (13)	8 (9)	19 (22)	9 (13)	
Primary specialty							<0.001
Anesthesiology, *n* (%)	234 (27)	186 (38)	7 (5)	7 (8)	32 (36)	2 (3)	
Intensive care, *n* (%)	495 (57)	270 (56)	54 (39)	65 (75)	41 (47)	65 (96)	
Internal medicine, *n* (%)	36 (4)	12 (2)	10 (7)	7 (8)	7 (8)	–	
Neurology, *n* (%)	70 (8)	5 (1)	57 (41)	5 (6)	3 (3)	–	
Pediatrics, *n* (%)	12 (1)	9 (2)	1 (1)	–	1 (1)	1 (1)	
Surgery, *n* (%)	21 (2)	3 (1)	11 (8)	3 (3)	4 (5)	–	
Years of practice							0.07
<5, *n* (%)	158 (18)	88 (18)	41 (29)	14 (16)	15 (17)	–	
5–10, *n* (%)	241 (28)	132 (27)	33 (24)	23 (26)	35 (40)	18 (26)	
11–15, *n* (%)	187 (22)	98 (20)	28 (20)	23 (26)	21 (24)	17 (25)	
16–20, *n* (%)	117 (13)	71 (15)	14 (10)	10 (11)	7 (8)	15 (22)	
21–25, *n* (%)	86 (10)	56 (12)	8 (6)	9 (10)	3 (3)	10 (15)	
>25, *n* (%)	79 (9)	40 (8)	16 (11)	8 (9)	7 (8)	8 (12)	
Type of ICU							<0.001
Neuro-ICU, *n* (%)	170 (20)	48 (10)	98 (70)	15 (17)	9 (10)	–	
Medical ICU, *n* (%)	46 (5)	20 (4)	–	9 (10)	15 (17)	2 (3)	
Surgical ICU, *n* (%)	75 (9)	54 (11)	7 (5)	3 (3)	11 (13)	–	
Mixed ICU, *n* (%)	550 (63)	346 (71)	33 (24)	58 (67)	51 (58)	62 (91)	
Pediatric ICU, *n* (%)	27 (3)	17 (4)	2 (1)	2 (3)	2 (2)	4 (6)	
Responsible for patients with ABI in the ICU							<0.001
Anesthesiologist, *n* (%)	76 (9)	67 (14)	1 (1)	–	8 (9)	–	
Intensivist, *n* (%)	494 (57)	311 (64)	57 (40)	43 (50)	26 (30)	57 (84)	
Neurologist, *n* (%)	13 (1)	2 (1)	5 (4)	2 (2)	4 (5)	–	
Neurosurgeon, *n* (%)	26 (3)	4 (1)	7 (5)	5 (6)	10 (11)	–	
Mixed responsibility, *n* (%)	259 (30)	101 (21)	70 (50)	37 (43)	40 (45)	11 (17)	
Number of available ICU beds (on the day of the survey)	14 [10–22]	12 [8–18]	20 [16–27]	13 [10–20]	15 [10–25]	18 [12–23]	<0.001
Hospital size (beds)							<0.001
<500, *n* (%)	345 (40)	175 (36)	40 (29)	65 (75)	39 (44)	26 (38)	
500–750, *n* (%)	208 (24)	107 (22)	39 (28)	16 (18)	18 (20)	28 (41)	
751–1000, *n* (%)	171 (20)	99 (20)	41 (29)	3 (3)	16 (18)	12 (18)	
>1000, *n* (%)	144 (17)	104 (21)	20 (14)	3 (3)	15 (17)	2 (3)	
Type of institution							<0.001
University, *n* (%)	341 (39)	205 (42)	84 (60)	12 (14)	25 (28)	15 (22)	
University-affiliated, *n* (%)	241 (28)	109 (22)	27 (19)	27 (31)	29 (33)	49 (72)	
Non-university-affiliated, *n* (%)	286 (33)	171 (35)	29 (21)	48 (55)	34 (39)	4 (6)	
Patients on the day of survey, *n* (%)	12 [8–19]	10 [8–16]	16 [12–20]	12 [8–18]	12 [10–24]	14 [10–21]	<0.001
Patients with ABI on day of survey, *n* (%)	3 [2–6]	3 [1–5]	10 [4–13]	4 [2–6]	4 [2–8]	3 [2–4]	<0.001

*ABI* Acute brain injury, ICU Intensive care unit

Data are presented as count (percent) or median [IQR]

### Transfusion thresholds

**Fig. 1:**
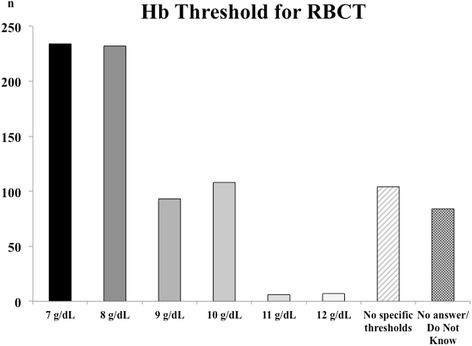
Number of respondents (*n*) for the recommended threshold of hemoglobin (Hb) used to initiate red blood cell transfusion (RBCT) in patients with acute brain injury

More than half the respondents (*n* = 466 [54%]) stated that an Hb threshold of 7 or 8 g/dl to initiate RBC transfusion in patients with acute brain injury is used in their ICUs (Fig. 1, Additional file 2: Table S1). The lowest proportion of respondents who reported that they initiated RBC transfusion for Hb thresholds less than or equal to 8 g/dl came from Europe and North America (both 49%), whereas in Oceania, this strategy was used by 69% of respondents. When only respondents providing a specific Hb threshold were considered, Central and South American respondents used a lower Hb threshold than did those from Europe; similarly, respondents from Oceania used a lower threshold than those from Europe and Asia/Africa (Additional file 2: Figure S2).

**Fig. 2:**
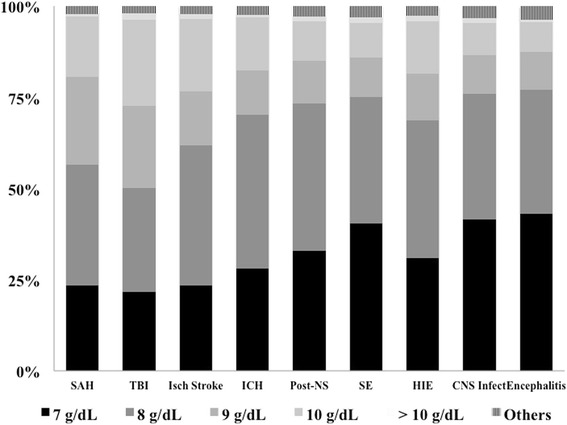
Proportion of respondents (%) for the different thresholds of hemoglobin used to initiate blood transfusion according to different cerebral pathologies. *SAH* Subarachnoid hemorrhage, *TBI* Traumatic brain injury, *Isch stroke* Ischemic stroke, *ICH* Intracranial hemorrhage, *NS* Neurosurgery *SE* Status epilepticus, *HIE* Hypoxic-ischemic encephalopathy, *CNS Infect* Infections of the central nervous system

A total of 435 (57%) of 764 of the respondents reported that they used a different Hb threshold for RBC transfusion in patients with acute brain injury following TBI (*n* = 349), SAH (*n* = 279), ischemic stroke (*n* = 181), and HIE (*n* = 109), whereas only a few considered that a different transfusion policy was needed for patients with ICH (*n* = 88), postneurosurgery (*n* = 88), seizure/SE (*n* = 33), CNS infections (*n* = 27), or encephalitis (*n* = 18). Respondents more frequently replied that an Hb concentration of 9 g/dl to initiate RBC transfusion was optimal in TBI (50%), SAH (43%), and ischemic stroke (38%) than in the setting of other conditions (Fig. 2, Table 2; Additional file 2: Table S1).

**Table 2:** Optimal hemoglobin threshold that respondents would use to initiate transfusion for different forms of acute brain injury

Optimal Hb threshold to initiate RBCT	SAH (*n* = 698)	TBI (*n* = 704)	IS (*n* = 694)	ICH (*n* = 693)	NS (*n* = 655)	Seizure/SE (*n* = 596)	HIE (*n* = 659)	CNS Inf (*n* = 594)	Enceph (*n* = 582)
7 g/dl, *n* (%)	163 (23)	152 (22)	163 (24)	195 (28)	216 (33)	241 (40)	203 (31)	247 (42)	250 (43)
8 g/dl, *n* (%)	232 (33)	200 (28)	266 (38)	291 (42)	264 (40)	206 (35)	250 (38)	204 (34)	199 (34)
9 g/dl, *n* (%)	167 (24)	159 (23)	103 (15)	85 (12)	77 (12)	65 (11)	84 (13)	63 (11)	60 (10)
10 g/dl, *n* (%)	116 (17)	167 (24)	138 (20)	100 (14)	71 (11)	57 (10)	94 (14)	52 (9)	47 (8)
>10 g/dl, *n* (%)	5 (1)	12 (2)	9 (1)	5 (1)	8 (1)	9 (2)	11 (2)	8 (1)	4 (1)
Others, *n* (%)	15 (2)	14 (2)	15 (2)	17 (2)	19 (3)	18 (3)	17 (3)	20 (3)	22 (4)

*Abbreviations: SAH* Subarachnoid hemorrhage, *TBI* Traumatic brain injury, *IS* Ischemic stroke, *ICH* Intracranial hemorrhage, *SE* Status epilepticus, *HIE* Hypoxic-ischemic encephalopathy, *CNS Infect* Infections of the central nervous system, *Enceph* Encephalitis, *Hb* Hemoglobin, *RBCT* Red blood cell transfusion

*p* < 0.001 for trend among groups

**Fig. 3:**
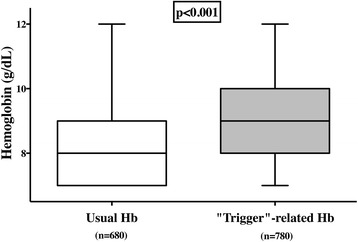
Median normal hemoglobin (Hb) values and altered values in the presence of trigger factors

Overall, noncerebral factors, such as known CAD (*n* = 474), active bleeding (*n* = 462), and low SvO_2_ (*n* = 393), were more often reported to influence the Hb threshold for transfusion than cerebral factors (Additional file 2: Table S1 and Figure S3). However, cerebral factors represented 40% of the potential determinants of RBC transfusion threshold change in North America, whereas this proportion was significantly lower on other continents, with the lowest value being in Oceania (17%) (Additional file 2: Figure S4). In the presence of any of these factors, 497 (57%) of 868 of respondents said they would increase the Hb threshold to initiate RBC transfusion to 9 or 10 g/dl (Additional file 2: Table S1and Figure S5). When only respondents providing a specific Hb threshold were considered, the Hb threshold to initiate transfusion was significantly higher when one or more influencing factors was present than when they were not (Fig. 3).

The main reason reported for giving a transfusion to a patient with acute brain injury was the potential increase in DO_2_ to ischemic regions (*n* = 625 [72%]) (Additional file 2: Table S1). Thirty percent of respondents said they would check the length of RBC storage before transfusion, but only one-third of these respondents would limit the administration of “old” RBC units. Only 76 of the respondents reported the maximum duration of RBC storage allowed for transfusion (median 15; range 2–150). Only 20% (*n* = 176) of respondents would give non-leukocyte-depleted RBC units to patients with acute brain injury.

The main reasons given for limiting transfusions in all patients with acute brain injury were the risk of transfusion-related acute lung injury (18%), risk of infection (17%), and altered immune response (14%) (Additional file 2: Table S1). More than 60% of respondents (*n* = 523) thought that a randomized clinical trial (RCT) comparing a restrictive and a liberal transfusion strategy was necessary in the setting of acute brain injury (Additional file 2: Figure S6); 306 (59%) respondents thought this study should compare two different Hb thresholds for initiating transfusion, and 217 (41%) thought it should compare a restrictive strategy with a strategy guided by neuromonitoring (e.g., PbtO_2_ or noninvasive cerebral oxygen saturation). The forms of acute brain injury that should be included in this study were, in particular, SAH (*n* = 592) and TBI (*n* = 560), whereas the need for such a study was considered less relevant for ischemic stroke (*n* = 354) or hemorrhagic stroke (*n* = 275), HIE (*n* = 211), postneurosurgery patients (*n* = 133), seizure/SE (*n* = 79), CNS infection (*n* = 79), or encephalitis (*n* = 66).

### Other issues

Few respondents measured iron levels before RBC transfusion in their clinical practice, except in North America and Asia/Africa, where more than 10% of those who responded stated that iron levels were checked in more than 50% of patients (i.e., frequently or often) (Additional file 2: Table S1). Three hundred three (41%) of 738 respondents (130 did not answer this question) gave iron supplements to patients with anemia who had acute brain injury (always, *n* = 17; only if the ICU length of stay was longer than 1 week, *n* = 43; only if iron levels were reduced, *n* = 200; only if patients received more than 8 RBC units, *n* = 14; only if no signs of infection, *n* = 29). Nearly 40% of respondents would frequently or often check for the presence of chronic anemia in patients with acute brain injury before initiating transfusion, with similar response rates across regions (Additional file 2: Table S1). More than 80% of respondents said they did not use erythropoietin (EPO) in patients with acute brain injury (Additional file 2: Table S1); among those who would give such therapy, EPO was used more in TBI (*n* = 83) and SAH (*n* = 62) than in other diseases (HIE, *n* = 46; ischemic stroke, *n* = 39; after neurosurgery, *n* = 25; ICH, *n* = 21; CNS infection or encephalitis, *n* = 18 each; seizure and/or SE, *n* = 10).

### Multivariable analysis

In multivariable analysis, the selection of Hb threshold to initiate RBC transfusion was significantly determined by regions (Asia/Africa and Oceania were significantly associated with a more conservative threshold when compared with Europe) and physicians’ characteristics (anesthesiologists were significantly more likely to use a liberal Hb threshold than were intensivists or physicians in intensivist-run ICUs) (Additional file 2: Table S2). Neurologists were more likely to state that noncerebral factors influenced their decision to transfuse than were intensivists, as were physicians working in large hospitals (750–1000 beds) compared with smaller hospitals.

Physicians working in ICUs in which patients with acute brain injuries were managed by a neurosurgeon more frequently used cerebral factors to trigger RBC transfusion than did physicians working in intensivist-run ICUs. The use of conservative transfusion policies, even if a trigger factor was present, was more common in physicians with more than 25 years of practice than in those with less than 5 years of practice and by physicians working in ICUs in which patients with acute brain injury were managed by a neurosurgeon compared with intensivist-run ICUs. Finally, physicians who said they used cerebral factors to alter transfusion thresholds were more likely to suggest that a randomized RCT should be performed to compare a restrictive strategy with a strategy guided by neuromonitoring rather than an RCT comparing two Hb thresholds.

## Discussion

This worldwide survey is the largest to provide data on the approach of physicians to anemia management and Hb thresholds for RBC transfusion in patients with acute brain injury. More than half of the respondents stated that they used an Hb threshold of 7–8 g/dl to initiate RBC transfusion after acute brain injury. A lower threshold for transfusion was observed in Africa/Asia and Oceania than in Europe. However, most respondents stated that they would increase the Hb trigger threshold in the presence of various factors, in particular CAD, active bleeding, and low SvO_2_. Most respondents said they felt an RCT was needed to compare different transfusion strategies in this patient population, in particular after SAH and TBI. Respondents’ characteristics that influenced transfusion policies in this setting included their origin, their background, and their experience, with the most liberal approaches used by respondents from Europe and North America, by anesthesiologists and neurologists/neurosurgeons, and by those with less than 5 years in practice.

Only two other surveys have evaluated transfusion practice in patients with acute brain injury. In the first study, Sena et al. [17] evaluated responses of 312 physicians, including trauma surgeons, neurosurgeons, and intensivists from level I trauma centers in the United States. The authors used two clinical scenarios, focusing only on TBI. Neurosurgeons reported the highest Hb thresholds for initiating RBC transfusion compared with trauma surgeons and intensivists in patients with normal (8.3 vs. 7.5 vs. 7.5 g/dl, respectively) and elevated (8.9 vs. 8.0 vs. 8.4 g/dl, respectively) intracranial pressure. Interestingly, fewer neurosurgeons than other physicians reported that they used systemic and cerebral factors to influence the decision to transfuse. In a second study, Kramer et al. [18] performed a survey of 282 academic neurointensivists, neurosurgeons, and multidisciplinary intensivists from the United States and Canada to evaluate transfusion policies for patients with SAH. The mean Hb concentration used to initiate RBC transfusion was higher in low-grade patients (8.2 g/dl) and in those with DCI (8.6 g/dl), with large variability in the proposed ranges. Again, neurosurgeons expressed the highest minimum Hb goals compared with other physicians. Respondents were more likely to transfuse patients with low PbtO_2_ values and high brain lactate-to-pyruvate ratios. Similarly, we also found that most of the respondents used an Hb threshold of 7–8 g/dl to initiate RBC transfusion in patients with acute brain injury, although nearly 60% of these respondents used a higher threshold in patients with TBI, SAH, and ischemic stroke.

Nevertheless, in our study, we made some interesting observations that were not reported in the previous surveys. First, Hb thresholds were more liberal among anesthesiologists than among intensivists. This may appear to be in contrast to recent guidelines from the American Society of Anesthesiologists suggesting the need for restrictive transfusion criteria to minimize RBC use with no additional risks for poor outcome or cardiac, neurological, or pulmonary complications [19]. Differences in practice between these two groups of physicians may explain these findings, with more than half of transfusions given by anesthesiologists partially initiated as a result of a physiological trigger (e.g., hypotension, tachycardia, preexisting anemia) rather than the absolute value of Hb [20]. Moreover, one study also showed that, in 48,086 surgical patients at a tertiary U.S. academic medical center, the target Hb value after transfusion ordered by an anesthesiologist was 11.7 ± 1.3 g/dl [21]. Second, previous surveys have been focused only on physicians in North America, whereas we report that a conservative (i.e., 7–8 g/dl) threshold of Hb was more often used in Asia/Africa and Oceania than in Europe to initiate transfusion. The lower threshold used in African and Asian countries may be due to limited resources and less blood availability, as well as to shortage of other supplies (e.g., bottles, bags) and greater risk of blood contamination [22]. The differences between Europe and Oceania may be explained by national and statewide initiatives in Australia/New Zealand to encourage implementation of patient blood management programs as a cost-effective standard of care in their public health system, whereas this approach is not entirely integrated into routine management in Europe [23, 24].

In this survey, we observed that many clinicians did not initiate RBC transfusion at a fixed Hb threshold, but adjusted their practice according to the presence of other factors. In particular, compared with intensivists, neurologists more frequently used noncerebral trigger factors, and neurosurgeons used more frequently used cerebral factors. The presence of systemic factors such as low SvO_2_ values has been widely used to trigger RBC transfusion in critically ill patients with sepsis [25]. RBC transfusion may also improve microvascular abnormalities associated with sepsis or may contribute to improved tissue oxygenation in patients with high lactate levels [26, 27]. Similarly, Oddo et al. [10] showed that only the combination of low Hb concentrations with reduced PbtO_2_ values negatively affected the outcome of patients with TBI. Thus, decisions regarding when to transfuse patients with acute brain injury remain difficult and should be titrated on an individual patient basis. Biomarkers of impaired systemic or tissue DO_2_ may help identify patients who are most likely to benefit from RBC transfusion in this setting. Heterogeneity in clinical practice among physicians with different backgrounds also suggests the need to better evaluate and identify which biomarkers could be used in this context.

More than 60% of respondents thought that an RCT comparing a restrictive and a liberal transfusion strategy was needed, in particular in patients with SAH and TBI. Interestingly, whereas 40% indicated that one group should be guided by neuromonitoring, only 13% of all respondents stated that neuromonitoring influenced their RBC transfusion policy and that transfusions were guided mainly by noncerebral events. This finding underlines that the availability of cerebral trigger factors as a tool to guide transfusion policy may be logistically difficult because these tools are not widely available. Also, the optimal threshold to initiate RBC transfusion in critically ill patients remains unclear, and many physicians still use predefined Hb values in their practice. Finally, it is also possible that the limited options provided in the survey format prevented respondents from providing other alternatives to an RCT comparing restrictive vs. liberal transfusion. Although the willingness to evaluate transfusion practices in the context of a randomized trial has also been suggested in previous surveys [17, 18], it remains unclear which threshold could accurately define a restrictive or a liberal approach. Current guidelines for RBC transfusion in the critically ill suggest initiating transfusion when the Hb is less than 7 g/dl because higher thresholds do not provide additional benefit [28]. Nevertheless, most practitioners would probably adopt a higher transfusion Hb threshold in patients with acute coronary syndrome, as well as in those at risk of secondary brain injury, such as patients with SAH with DCI or in patients with severe TBI [11, 29, 30]. However, it remains unclear whether using RBC transfusion to increase Hb concentrations to 9–11 g/dl is a logical therapeutic decision to improve cerebral oxygenation and neurological recovery in these patients. Indeed, in patients with TBI, the increase in PbtO_2_ after RBC transfusion was generally limited [31, 32]. In patients with low-grade SAH, each 1.0 g/dl increase in Hb concentration after transfusion was associated with an increase of 1.4 mmHg in PbtO_2_, without significant effects on cerebral metabolism [33]. In another study, RBC transfusion resulted in a significant improvement in cerebral DO_2_, in particular in those cerebral territories with the lowest baseline DO_2_, and these effects were more significant than those produced by fluid expansion or vasopressors [34, 35].

Although we observed some differences in Hb thresholds and RBC transfusion practices across regions and specialties, we could not determine from our survey whether this was due to discrepancies in local protocols, lack of compliance with general recommendations, or other factors that we did not investigate. For example, we showed that working in large hospitals (for the use of noncerebral triggers) and a greater degree of seniority (for the use of a conservative threshold) may influence the decision to transfuse. Whether these observations reflect a different familiarity with published literature on these issues or just personal practice is difficult to determine.

This survey has several limitations that need to be acknowledged. First, the validity of the survey depends on a high response rate among target physicians. Because the questionnaire was distributed using email addresses of members of different medical societies, we could not determine the potential denominator, which may have resulted in an underpowered analysis. The total number of potential respondents (i.e., considering the members of participating critical care societies; a list of respondents per countries is provided in Additional file 1: Appendix 2) probably exceeded 15,000 people, and this might significantly limit the external validity/generalizability of our findings. We did not specifically contact only physicians who were experts in neurocritical care, as was the case in previous surveys [17, 18]; we also allowed other physicians working in general or mixed ICUs treating these patients to respond, and this may provide a broader picture of the general management of these patients who are admitted to nonspecialized ICUs in many countries. Second, survey development and testing should follow a process of item generation through a process of a systematic review; item reduction; pretesting; and pilot testing for clinical sensibility, reliability, and validity [36]. Our survey was based on a consensus within experts in the Neuro-Intensive Care Section of the ESICM, and the preparation process might have affected comprehensiveness, clarity, and face validity as well as respondents’ interpretation of questions. Third, as is true for all surveys, the responses do not necessarily reflect local practice but personal opinions of the strategy used to manage RBC transfusion in this setting. A prospective audit collecting data on this topic would be welcome to provide more real-world data for this therapeutic intervention. Fourth, we had few respondents from Africa and needed to consider Asia and Africa as a single group, although practices may be different in these two areas. Fifth, our survey was conducted before the publication of a recent U.S. study evaluating RBC transfusion management in patients with TBI [37], which showed no differences between a liberal (Hb less than 10 g/dl) and a restrictive (Hb less than 7 g/dl) transfusion strategy. It is impossible to know whether these results would have influenced the responses in our survey. Finally, because the survey was conducted anonymously, we could not control whether several respondents worked at the same center, which may have influenced the variability and generalizability of the results.

## Conclusions

In this large survey, most physicians reported using a conservative Hb (7–8 g/dl) to initiate RBC transfusion in patients with acute brain injury, although half of them would use a higher Hb threshold after TBI, SAH, and ischemic stroke. Both systemic and cerebral factors may influence the choice of Hb threshold used for transfusion in these patients. Several differences according to physician specialty and regional areas were identified. An RCT comparing two different Hb thresholds for RBC transfusion in this setting would be more than welcome. Whether the optimal trial design should rely on predefined Hb values to separate the study groups or use specific thresholds (either cerebral or systemic) remains to be further clarified.

## Additional files

Additional file 1: **Appendix 1.** Survey questionnaire. **Appendix 2:** Number of respondents per countries. (DOCX 37 kb)

Additional file 2: **Table S1.** Transfusion policies among respondents. **Table S2.** Multivariable logistic regression analyses assessing associations between respondent characteristics and various transfusion policies for patients with acute brain injury (ABI). **Figure S1.** Regional distribution of respondents across continents. **Figure S2.** Median threshold of hemoglobin (Hb) recommended to initiate blood transfusion in patients with ABI in different geographic regions. **Figure S3.** Number of respondents reporting that different trigger factors (*n*) would influence their decision to initiate blood transfusion in patients with ABI. **Figure S4.** Relative proportions of noncerebral and cerebral trigger factors (%) used to initiate blood transfusion in patients with ABI, according to geographic region. **Figure S5.** Number of respondents (*n*) stating they would use a new threshold of hemoglobin (Hb) to initiate blood transfusion in patients with ABI in the presence of a trigger factor. **Figure S6.** Number of respondents (*n*) agreeing with the need for a randomized clinical trial (RCT) in patients with anemia from patients with ABI. (DOCX 884 kb)
