# Supplementary material for: Hemoglobin concentrations and RBC transfusion thresholds in patients with acute brain injury: an international survey
Source: Crit Care. 2017 Jun 17;21:159. doi: 10.1186/s13054-017-1748-4 (PMC5473997; doi:10.1186/s13054-017-1748-4)
Supplement: Supplementary file 1 — Appendix 1. Survey questionnaire. Appendix 2: Number of respondents per countries. (DOCX 37 kb) [file 13054_2017_1748_MOESM1_ESM.docx]

**Additional file 1**

**Hemoglobin concentrations and RBC transfusion thresholds in patients with acute brain injury:**

**An International Survey**

***Rafael BADENES^1^, Mauro ODDO^2^, José I. SUAREZ^3^, Massimo ANTONELLI^4^, Jeffrey LIPMAN^5^, Giuseppe CITERIO^6^,***

***Fabio Silvio TACCONE^7^***

*^1^Department of Anesthesiology and Surgical Intensive Care*

*Hospital Clinic Universitari*

*Valencia, Spain*

[*rafaelbadenes@gmail.com*](mailto:rafaelbadenes@gmail.com)

*^2^Department of Intensive Care Medicine*

*Centre Hospitalier Universitaire Vaudois (CHUV)*

*Lausanne University Hospital,*

*1011 Lausanne, Switzerland*

[*Mauro.Oddo@chuv.ch*](mailto:Mauro.Oddo@chuv.ch)

*^3^Division of Vascular Neurology and Neurocritical Care*

*Department of Neurology*

*Baylor College of Medicine*

*CHI Baylor St Luke’s Medical Center*

*Houston, TX*

[*jisuarez@bcm.edu*](mailto:jisuarez@bcm.edu)

*^4^Department of Anesthesiology and Intensive Care Medicine*

*Catholic University-Fondazione Policlinico A.Gemelli University Hospital,*

*Rome, Italy*

[*m.antonelli@rm.unicatt.it*](mailto:m.antonelli@rm.unicatt.it)

*^5^Intensive Care Services*

*Royal Brisbane and Womens Hospital*

*Burns Trauma Critical Care Research Centre*

*University of Queensland*

*Australia*

[*j.lipman@uq.edu.au*](mailto:j.lipman@uq.edu.au)

*^6^School of Medicine and Surgery*

*University of Milano-Bicocca*

*Neurointensive Care, San Gerardo Hospital*

*ASST-Monza, Italy*

[*giuseppe.citerio@unimib@it*](mailto:giuseppe.citerio@unimib@it)

*^7^Department of Intensive Care*

*Erasme Hospital, Université Libre de Bruxelles*

*Route de Lennik, 808 – 1070, Brussels, Belgium*

[*ftaccone@ulb.ac.be*](mailto:ftaccone@ulb.ac.be)

**Word count:** 4070 (Abstract: 238 – References:)

**Running head:** Hemoglobin and Brain Injury

**Keywords:** Hemoglobin, transfusion, threshold, brain injury, outcome

*Correspondence*: Pr. **Fabio Silvio TACCONE**

Department of Intensive Care

Erasme Hospital

Université Libre de Bruxelles (ULB)

Route de Lennik, 808

1070 – Brussels (BELGIUM)

email: ftaccone@ulb.ac.be

tel : +322 555 5587

fax : +322 555 469

**A Survey on Hemoglobin levels and RBC transfusions thresholds**

**in patients with acute brain injury**

**APPENDIX 1**

**Responder information :**

1. **In which country do you practice?**
2. **Have you a specific certification in neuro-critical care?**

- Yes, only in neurocritical care
- Yes, both neurocritical and critical care
- No, only critical care
- No

1. **Your primary specialty is:**

- Intensive Care
- Internal Medicine
- Anesthesiology
- Neurology
- Surgery (including Neurosurgery)
- Pediatrics
- Others

1. **Number of years of practice in critical care**

- < 5
- 5-10
- 11-15
- 16-20
- 21-25
- > 25

1. **Type of Intensive Care Unit (ICU) :**

- Neuro-ICU
- Medical ICU
- Surgical ICU
- Mixed ICU
- Pediatric ICU
- Others

1. **Who is responsible for the care of patients with acute brain injury in your ICU: (more than one = “mixed responsibility”)**

- Intensivist (so-called « closed ICU »)
- Anesthesiologist
- Neurosurgeon
- Neurologist
- Others

1. **Number of available ICU beds :**
2. **Hospital size:**

- < 500 beds
- 50-750 beds
- 750-1000 beds
- > 1000 beds

1. **Institution :**

- University
- University affiliate
- Non-university

**Patient information :**

1. **Primary brain injuries admitted to your ICU (tick all that apply):**

- subarachnoid hemorrhage
- traumatic brain injury
- ischemic stroke
- hemorrhagic stroke
- post-operative neurosurgical patients
- status epilepticus (convulsive or non-convulsive)
- post-anoxic coma
- central nervous system (CNS) infections
- autoimmune or inflammatory encephalitis

1. **Number of patients in your ICU today is:**
2. **Number of patients with primary brain injury (see list at question 10, EXCLUDING programmed neurosurgery) in your ICU today:**
3. **Number of patients after programmed neurosurgery in your ICU today:**
4. **Recommended Hb threshold for red blood cell (RBC) transfusion in patients with primary brain injury at your center is:**

- 7 g/dL
- 8 g/dL
- 9 g/dL
- 10 g/dL
- 11 g/dL
- 12 g/dL
- Other: _____ g/dL
- no Hb threshold for RBC transfusion

**RBC and transfusion strategy with regard to the primary brain injury**

1. **Do you think Hb thresholds for RBC transfusion differ among the various primary brain injuries:**

- Yes
- No

1. **If YES, which of the following diseases would require higher Hb levels in your opinion (tick all that apply):**

- subarachnoid hemorrhage
- traumatic brain injury
- ischemic stroke
- hemorrhagic stroke
- post-operative neurosurgical patients
- status epilepticus (convulsive and non-convulsive)
- post-anoxic coma
- central nervous system (CNS) infections
- autoimmune or inflammatory encephalitis

1. **What is the Hb threshold you would recommend in these pathologies requiring higher Hb levels?** (please indicate the new Hb levels only for the primary brain injuries selected at question 16)

subarachnoid hemorrhage

- 7 g/dL
- 8 g/dL
- 9 g/dL
- 10 g/dL
- 11 g/dL
- 12 g/dL
- Other: _____ g/dL

traumatic brain injury

- 7 g/dL
- 8 g/dL
- 9 g/dL
- 10 g/dL
- 11 g/dL
- 12 g/dL
- Other: _____ g/dL

ischemic stroke

- 7 g/dL
- 8 g/dL
- 9 g/dL
- 10 g/dL
- 11 g/dL
- 12 g/dL
- Other: _____ g/dL

hemorrhagic stroke

- 7 g/dL
- 8 g/dL
- 9 g/dL
- 10 g/dL
- 11 g/dL
- 12 g/dL
- Other: _____ g/dL

post-operative neurosurgical patients

- 7 g/dL
- 8 g/dL
- 9 g/dL
- 10 g/dL
- 11 g/dL
- 12 g/dL
- Other: _____ g/dL

status epilepticus (convulsive and non-convulsive)

- 7 g/dL
- 8 g/dL
- 9 g/dL
- 10 g/dL
- 11 g/dL
- 12 g/dL
- Other: _____ g/dL

post-anoxic coma

- 7 g/dL
- 8 g/dL
- 9 g/dL
- 10 g/dL
- 11 g/dL
- 12 g/dL
- Other: _____ g/dL

central nervous system (CNS) infections

- 7 g/dL
- 8 g/dL
- 9 g/dL
- 10 g/dL
- 11 g/dL
- 12 g/dL
- Other: _____ g/dL

autoimmune or inflammatory encephalitis

- 7 g/dL
- 8 g/dL
- 9 g/dL
- 10 g/dL
- 11 g/dL
- 12 g/dL
- Other: _____ g/dL

1. **What is the Hb threshold you actually use in these pathologies?**

subarachnoid hemorrhage

- 7 g/dL
- 8 g/dL
- 9 g/dL
- 10 g/dL
- 11 g/dL
- 12 g/dL
- Other: _____ g/dL

traumatic brain injury

- 7 g/dL
- 8 g/dL
- 9 g/dL
- 10 g/dL
- 11 g/dL
- 12 g/dL
- Other: _____ g/dL

ischemic stroke

- 7 g/dL
- 8 g/dL
- 9 g/dL
- 10 g/dL
- 11 g/dL
- 12 g/dL
- Other: _____ g/dL

hemorrhagic stroke

- 7 g/dL
- 8 g/dL
- 9 g/dL
- 10 g/dL
- 11 g/dL
- 12 g/dL
- Other: _____ g/dL

post-operative neurosurgical patients

- 7 g/dL
- 8 g/dL
- 9 g/dL
- 10 g/dL
- 11 g/dL
- 12 g/dL
- Other: _____ g/dL

status epilepticus (convulsive and non-convulsive)

- 7 g/dL
- 8 g/dL
- 9 g/dL
- 10 g/dL
- 11 g/dL
- 12 g/dL
- Other: _____ g/dL

post-anoxic coma

- 7 g/dL
- 8 g/dL
- 9 g/dL
- 10 g/dL
- 11 g/dL
- 12 g/dL
- Other: _____ g/dL

central nervous system (CNS) infections

- 7 g/dL
- 8 g/dL
- 9 g/dL
- 10 g/dL
- 11 g/dL
- 12 g/dL
- Other: _____ g/dL

autoimmune or inflammatory encephalitis

- 7 g/dL
- 8 g/dL
- 9 g/dL
- 10 g/dL
- 11 g/dL
- 12 g/dL
- Other: _____ g/dL

**Complementary questions and therapies**

1. **Do you check iron levels in patients with primary acute brain injury before starting transfusions?**

- Yes
- Often (>75% of patients)
- Frequently (50-75% of patients)
- Sometimes (25-50% of patients)
- Seldom (<25% of patients)
- Never

1. **Do you give iron supplements to anemic patients with primary acute brain injury?**

- Always
- Only if iron levels are reduced
- Only if ICU stay is longer than one week
- Only if patient has been transfused for more than eight red blood cells packs
- Only if patient has no signs of infection
- Never

1. **Do you check if anemic patients with primary acute brain injury suffered from chronic anemia before ICU admission?**

- Always
- Often (>75% of patients)
- Frequently (50-75% of patients)
- Sometimes (25-50% of patients)
- Seldom (<25% of patients)
- Never

1. **Do you give erythropoietin to anemic patients with acute primary brain injury?**

- Yes, to increase Hb levels if a chronic anemia was present before ICU admission
- Yes, but I give erythropoietin to all anemic patients with acute brain injury, regardless of their chronic Hb levels
- Only if patient received erythropoietin before ICU admission
- I give erythropoietin as neuroprotective therapy in those patients
- No

1. **If YES, do you think that erythropoietin would be more suitable for a specific type of primary brain injury (tick all that apply) :**

- subarachnoid hemorrhage
- traumatic brain injury
- ischemic stroke
- hemorrhagic stroke
- post-operative neurosurgical patients
- status epilepticus (convulsive and non-convulsive)
- post-anoxic coma
- central nervous system (CNS) infections
- autoimmune or inflammatory encephalitis

**RBC and transfusion strategy with regard to clinical situations**

1. **Do any of the below situations modify your Hb threshold for transfusion (tick all that apply):**

- age
- active bleeding
- Glasgow Coma Score ≤ 8
- delayed cerebral ischemia
- intracranial pressure > 20 mmHg
- brain tissue PO2 < 15 mmHg
- coronary artery disease
- mixed (SvO2) or central (SvcO2) venous saturation < 65% in the absence of hypovolemia
- blood lactate > 2.5 mmol/L
- other neuro-monitoring devices (including neuroimaging) suggesting cerebral ischemia
- others : ___________________
- no change in threshold

1. **If YES, what would then be your new Hb threshold? (tick the new Hb threshold you would apply):**

- 7 g/dL
- 8 g/dL
- 9 g/dL
- 10 g/dL
- 11 g/dL
- 12 g/dL

- I would target it to brain physiological variables (such as PbO2, NIRS, microdialysis etc.)

- Other: _____ g/dL

1. **What would be the reason to change the threshold for RBC transfusion? (tick all that apply):**

- to increase cerebral oxygenation
- to increase cerebral blood flow
- to specifically improve oxygen delivery in ischemic regions
- volume expansion and increased cardiac output
- others : _____________________________

1. **Do you check the number of days of RBC storage?**

- Yes, but I don’t use any limit of storage to give them
- Yes, and I don’t give these packs after ____ days of storage
- Never

1. **Do you give RBC that are leukocyte depleted ?**

- Yes, always
- Yes, if available
- Don’t know
- No

**Liberal vs. Restrictive strategy for RBC administration**

1. **What is the main reason to limit a liberal transfusion strategy in patients with primary brain injury? (tick all that apply)**

- Allergy
- Alloimmunization
- Alter blood viscosity/rheology and negatively affect microcirculation
- Alter coagulation
- Altered immune response
- Fluid overload
- Increased infections
- Ionic imbalance (Calcium and Potassium)
- Iron overload
- Transfusion-related acute lung injury (TRALI)
- Others : _____________________________

1. **Do you think a large multicenter randomized trial comparing the effects of a restrictive (i.e. 7 g/dL) vs. a liberal (i.e. 9-10 g/dL) RBC transfusion strategy on the outcome of patients with primary brain injury is needed ?**

- Yes and it should compare two different Hb thresholds for RBC transfusion
- Yes but it should compare a restrictive strategy to another guided by neuromonitoring (such as PbO2, NIRS, microdialysis etc.)
- No, I think that it would be unethical to use a restrictive RBC transfusion strategy in this population
- No, I think that the use of a liberal RBC transfusion strategy should be avoided for the progressive reduction of RBC availability in different countries
- No, I do no think that Hb levels have an impact on the outcome of patients with primary brain injury
- Others : ________________________________________________________

1. **If YES, is there a particular subgroup of patients with primary brain injury that needs to be studied by such a trial? (tick all that apply)**

- subarachnoid hemorrhage
- traumatic brain injury
- ischemic stroke
- hemorrhagic stroke
- post-operative neurosurgical patients
- status epilepticus (convulsive and non-convulsive)
- post-anoxic coma
- central nervous system (CNS) infections
- autoimmune or inflammatory encephalitis

**Appendix 2.** Number of respondents per countries.

Italy 133

USA 129

UK 60

Switzerland 58

Australia 57

Brazil 52

India 30

Germany 29

Spain 27

France 26

Belgium 22

Greece 16

Sweden 16

Netherlands 15

Saudi Arabia 12

new Zealand 11

Portugal 11

Argentine 9

Canada 9

Ireland 9

Russia 8

Austria 7

Mexico 7

Turkey 7

Denmark 6

Finland 6

Peru 5

Colombia 4

Czech Republic 4

Egypt 4

Georgia 4

Hungary 4

Indonesia 4

Romania 4

Singapore 4

Slovenia 4

Japon 3

Norway 3

Panama 3

UAE 3

Bolivia 2

Israel 2

Lithuania 2

Qatar 2

Slovakia 2

Taiwan 2

Vietnam 2

Bangladesh 1

Bosnia 1

Bulgaria 1

Chile 1

China 1

Costarica 1

Cyprus 1

Guadeloupe 1

Guatemala 1

Iran 1

South Korea 1

Lebanon 1

Luxembourg 1

Malta 1

Marocco 1

Oman 1

Pakistan 1

Philippines 1

Poland 1

Puerto Rico 1

San Marino 1

Serbia 1

South Africa 1

Sri Lanka 1

Sudan 1

Syria 1

Ukraina 1

Uruguay 1

Yemen 1
