# Supplementary material for: Hemoglobin concentrations and RBC transfusion thresholds in patients with acute brain injury: an international survey
Source: Crit Care. 2017 Jun 17;21:159. doi: 10.1186/s13054-017-1748-4 (PMC5473997; doi:10.1186/s13054-017-1748-4)

**Additional file 2**

**Hemoglobin concentrations and RBC transfusion thresholds in patients with acute brain injury:**

**An International Survey**

***Rafael BADENES^1^, Mauro ODDO^2^, José I. SUAREZ^3^, Massimo ANTONELLI^4^, Jeffrey LIPMAN^5^, Giuseppe CITERIO^6^,***

***Fabio Silvio TACCONE^7^***

*^1^Department of Anesthesiology and Surgical Intensive Care*

*Hospital Clinic Universitari*

*Valencia, Spain*

[*rafaelbadenes@gmail.com*](mailto:rafaelbadenes@gmail.com)

*^2^Department of Intensive Care Medicine*

*Centre Hospitalier Universitaire Vaudois (CHUV)*

*Lausanne University Hospital,*

*1011 Lausanne, Switzerland*

[*Mauro.Oddo@chuv.ch*](mailto:Mauro.Oddo@chuv.ch)

*^3^Division of Vascular Neurology and Neurocritical Care*

*Department of Neurology*

*Baylor College of Medicine*

*CHI Baylor St Luke’s Medical Center*

*Houston, TX*

[*jisuarez@bcm.edu*](mailto:jisuarez@bcm.edu)

*^4^Department of Anesthesiology and Intensive Care Medicine*

*Catholic University-Fondazione Policlinico A.Gemelli University Hospital,*

*Rome, Italy*

[*m.antonelli@rm.unicatt.it*](mailto:m.antonelli@rm.unicatt.it)

*^5^Intensive Care Services*

*Royal Brisbane and Womens Hospital*

*Burns Trauma Critical Care Research Centre*

*University of Queensland*

*Australia*

[*j.lipman@uq.edu.au*](mailto:j.lipman@uq.edu.au)

*^6^School of Medicine and Surgery*

*University of Milano-Bicocca*

*Neurointensive Care, San Gerardo Hospital*

*ASST-Monza, Italy*

[*giuseppe.citerio@unimib@it*](mailto:giuseppe.citerio@unimib@it)

*^7^Department of Intensive Care*

*Erasme Hospital, Université Libre de Bruxelles*

*Route de Lennik, 808 – 1070, Brussels, Belgium*

[*ftaccone@ulb.ac.be*](mailto:ftaccone@ulb.ac.be)

**Word count:** 4070 (Abstract: 238 – References:)

**Running head:** Hemoglobin and Brain Injury

**Keywords:** Hemoglobin, transfusion, threshold, brain injury, outcome

*Correspondence*: Pr. **Fabio Silvio TACCONE**

Department of Intensive Care

Erasme Hospital

Université Libre de Bruxelles (ULB)

Route de Lennik, 808

1070 – Brussels (BELGIUM)

email: ftaccone@ulb.ac.be

tel : +322 555 5587

fax : +322 555 4698

**Additional file 2: Table S1.** Transfusion policies among respondents. Data are presented as counts (percentage) or median [ranges].

|  | **Overall**  **(n=868)** | **Europe**  **(n=485)** | **North America**  **(n=140)** | **Central & South America**  **(n=87)** | **Asia/Africa**  **(n=88)** | **Oceania**  **(n=68)** | **p value** |
| --- | --- | --- | --- | --- | --- | --- | --- |
| **Recommended Hb threshold used to initiate RBCT** |  |  |  |  |  |  | <0.001 |
| *7 g/dL, n (%)* | 234 (27) | 108 (22) | 41 (29) | 34 (39) | 23 (26) | 28 (41) |  |
| *8 g/dL, n (%)* | 232 (27) | 131 (27) | 27 (19) | 22 (26) | 33 (37) | 19 (28) |  |
| *9 g/dL, n (%)* | 93 (10) | 65 (13) | 7 (5) | 8 (9) | 10 (11) | 3 (4) |  |
| *10 g/dL, n (%)* | 108 (12) | 66 (13) | 16 (11) | 9 (10) | 14 (16) | 3 (4) |  |
| *11 g/dL, n (%)* | 6 (1) | 5 (1) | 1 (1) | - | - | - |  |
| *12 g/dL, n (%)* | 7 (1) | 6 (1) | - | 1 (1) | - | - |  |
| *No specific threshold, n (%)* | 104 (12) | 48 (10) | 30 (21) | 9 (10) | 5 (6) | 12 (18) |  |
| *Don’t know – No Answer, n (%)* | 84 (10) | 56 (12) | 18 (13) | 4 (5) | 3 (3) | 3 (4) |  |
|  |  |  |  |  |  |  |  |
| **Factors that influence RBCT policy** |  |  |  |  |  |  | <0.001 |
| ***Non-cerebral factors*** |  |  |  |  |  |  |  |
| *Active bleeding, n (%)* | 462 (53) | 217 (45) | 86 (61) | 52 (60) | 61 (69) | 46 (68) |  |
| *CAD, n (%)* | 474 (54) | 271 (56) | 64 (46) | 48 (55) | 52 (59) | 39 (57) |  |
| *Low SvO_2_, n (%)* | 393 (45) | 225 (46) | 61 (44) | 48 (55) | 42 (48) | 17 (25) |  |
| *Age, n (%)* | 295 (34) | 174 (36) | 38 (27) | 23 (26) | 30 (34) | 30 (44) |  |
| *Lactate > 2.5 mEq/L, n (%)* | 282 (32) | 167 (34) | 45 (32) | 21 (24) | 34 (39) | 15 (22) |  |
|  |  |  |  |  |  |  |  |
| ***Cerebral factors*** |  |  |  |  |  |  |  |
| *PbtO2 < 15 mmHg, n (%)* | 314 (36) | 169 (34) | 75 (54) | 32 (37) | 27 (31) | 11 (16) |  |
| *DCI, n (%)* | 231 (27) | 115 (24) | 65 (46) | 19 (22) | 21 (24) | 11 (16) |  |
| *Neuromonitoring, n (%)* | 117 (13) | 63 (13) | 32 (23) | 11 (13) | 7 (8) | 4 (6) |  |
| *ICP>20 mmHg, n (%)* | 82 (9) | 53 (11) | 12 (9) | 6 (7) | 10 (11) | 1 (1) |  |
| *GCS < 9, n (%)* | 102 (12) | 70 (25) | 12 (9) | 4 (5) | 13 (15) | 3 (4) |  |
| *None of those, n (%)* | 32 (4) | 21 (4) | 2 (1) | 3 (4) | 2 (2) | 4 (6) |  |
|  |  |  |  |  |  |  |  |
| **New Hb threshold for RBCT if trigger factors were present** |  |  |  |  |  |  | 0.99 |
| *7 g/dL, n (%)* | 19 (2) | 10 (2) | 3 (2) | 2 (2) | 3 (3) | 1 91) |  |
| *8 g/dL, n (%)* | 246 (28) | 135 (28) | 40 (29) | 24 (28) | 32 (36) | 15 (22) |  |
| *9 g/dL, n (%)* | 199 (23) | 114 (23) | 27 (19) | 18 (21) | 20 (23) | 20 (29) |  |
| *10 g/dL, n (%)* | 298 (34) | 161 (33) | 54 (39) | 33 (38) | 24 (27) | 26 (38) |  |
| *11 g/dL, n (%)* | 10 (1) | 5 (1) | 2 (1) | 2 (2) | - | 1 (1) |  |
| *12 g/dL, n (%)* | 8 (1) | 3 (1) | 1 (1) | 1 (1) | 2 (2) | 1 (1) |  |
| *Guided by neuromonitoring, n (%)* | 71 (8) | 47 (10) | 11 (8) | 4 (5) | 6 (7) | 3 (4) |  |
| *No answer, n (%)* | 17 (2) | 10 (2) | 2 (1) | 3 (3) | 1 (1) | 1 (1) |  |
|  |  |  |  |  |  |  |  |
| **What would be the reason to change the threshold for RBCT?** |  |  |  |  |  |  | 0.99 |
| *To increase cerebral oxygenation, n (%)* | 372 (42) | 215 (44) | 60 (43) | 33 (38) | 39 (44) | 25 (37) |  |
| *To increase CBF, n (%)* | 106 (12) | 61 (12) | 20 (14) | 8 (9) | 9 (10) | 8 (12) |  |
| *To improve DO_2_ in ischemic regions, n (%)* | 625 (84) | 340 (70) | 107 (76) | 63 (72) | 63 (72) | 52 (76) |  |
| *Volume expansion and increased CO, n (%)* | 366 (42) | 203 (42) | 61 (44) | 33 (38) | 39 (44) | 30 (44) |  |
|  |  |  |  |  |  |  |  |
| **Check the duration of RBC storage?** |  |  |  |  |  |  |  |
| *Yes, but not used to limit RBCT, n (%)* | 198 (22) | 100 (21) | 36 (26) | 16 (19) | 25 (28) | 21 (31) | 0.27 |
| *Yes, and limit the use of “old” RBCT, n (%)* | 75 (8) | 42 (9) | 12 (9) | 8 (10) | 5 (6) | 8 (12) |  |
| *Never, n (%)* | 565 (65) | 324 (67) | 88 (63) | 58 (67) | 58 (66) | 37 (54) |  |
| *No answer, n (%)* | 30 (3) | 19 (4) | 4 (3) | 5 (6) | - | 2 (3) |  |
|  |  |  |  |  |  |  |  |
| *Maximum days of storage to give RBCT* | 15 [2-120] | 15 [3-120] | 7 [2-30] | 15 [2-28] | 17 [5-28] | 14 [4-28] | 0.07 |
|  |  |  |  |  |  |  |  |
| **Give leukocyte-depleted RBCT?** |  |  |  |  |  |  |  |
| *Yes - always, n (%)* | 323 (37) | 182 (28) | 46 (33) | 32 (37) | 30 (34) | 33 (49) | 0.87 |
| *Yes – if available, n (%)* | 162 (19) | 84 (17) | 32 (23) | 14 (16) | 19 (22) | 13 (19) |  |
| *No, n (%)* | 176 (20) | 99 (20) | 27 (19) | 19 (22) | 19 (22) | 12 (18) |  |
| *Don’t know, n (%)* | 207 (24) | 120 (25) | 35 (25) | 22 (25) | 20 (23) | 10 (15) |  |
|  |  |  |  |  |  |  |  |
| **Main reason(s) to limit RBCT** |  |  |  |  |  |  | 0.9 |
| *Allergy, n (%)* | 227 (26) | 126 (26) | 41 (29) | 14 (16) | 29 (33) | 17 (25) |  |
| *Alloimmunization, n (%)* | 251 (29) | 131 (27) | 48 (34) | 23 (26) | 31 (35) | 18 (26) |  |
| *Altered viscosity and impaired MC, n (%)* | 323 (37) | 180 (37) | 54 (39) | 29 (33) | 30 (34) | 30 (44) |  |
| *Altered coagulation, n (%)* | 260 (30) | 142 (29) | 50 (36) | 20 (23) | 31 (35) | 17 (25) |  |
| *Altered immune response, n (%)* | 378 (43) | 209 (43) | 58 (41) | 38 (44) | 35 (40) | 38 (56) |  |
| *Fluid overload, n (%)* | 242 (28) | 137 (28) | 47 (34) | 15 (17) | 21 (24) | 22 (32) |  |
| *Increased risk of infection, n (%)* | 487 (56) | 246 (51) | 96 (69) | 50 (75) | 51 (61) | 44 (65) |  |
| *Ionic imbalance, n (%)* | 67 (8) | 34 (7) | 15 (3) | 5 (6) | 8 (9) | 5 (7) |  |
| *Iron overload, n (%)* | 65 (8) | 33 (7) | 12 (2) | 7 (8) | 7 (8) | 4 (6) |  |
| *TRALI, n (%)* | 495 (57) | 304 (63) | 70 (14) | 46 (52) | 40 (53) | 35 (51) |  |
|  |  |  |  |  |  |  |  |
| **Check for iron levels before RBCT? *** |  |  |  |  |  |  | 0.0007 |
| *Never, n (%)* | 397 (46) | 231 (48) | 41 (30) | 55 (63) | 37 (42) | 33 (49) |  |
| *Seldom, n (%)* | 169 (19) | 81 (16) | 35 (25) | 12 (14) | 20 (23) | 21 (31) |  |
| *Sometimes, n (%)* | 104 (12) | 63 (13) | 20 (14) | 6 (7) | 10 (11) | 5 (7) |  |
| *Frequently, n (%)* | 38 (4) | 23 (5) | 7 (5) | 2 (2) | 5 (6) | 1 (1) |  |
| *Often, n (%)* | 50 (6) | 21 (4) | 19 (14) | 2 (2) | 8 (9) | - |  |
| *No answer, n (%)* | 110 (13) | 66 (14) | 18 (13) | 10 (11) | 8 (9) | 8 (12) |  |
|  |  |  |  |  |  |  |  |
| **Check for chronic anemia before transfusion *** |  |  |  |  |  |  | 0.45 |
| *Never, n (%)* | 128 (15) | 65 (13) | 14 (10) | 20 (23) | 15 (17) | 14 (21) |  |
| *Seldom, n (%)* | 153 (18) | 90 (19) | 22 (16) | 10 (11) | 20 (23) | 11 (16) |  |
| *Sometimes, n (%)* | 59 (7) | 30 (6) | 8 (6) | 10 (11) | 6 (7) | 5 (7) |  |
| *Frequently, n (%)* | 58 (7) | 35 (7) | 14 (10) | 4 (5) | 4 (5) | 1 (1) |  |
| *Often, n (%)* | 290 (33) | 158 (33) | 55 (39) | 26 (30) | 27 (31) | 24 (35) |  |
| *No answer, n (%)* | 180 (21) | 107 (22) | 27 (19) | 17 (20) | 16 (18) | 13 (19) |  |
|  |  |  |  |  |  |  |  |
| **Give EPO to anemic patients with ABI** |  |  |  |  |  |  | 0.89 |
| *Yes, as a neuroprotective drug, n (%)* | 7 (1) | 2 (1) | 2 (1) | - | 3 (3) | - |  |
| *Yes, to all ABI anemic patients, n (%)* | 14 (2) | 7 (1) | 2 (1) | 2 (2) | 2 (2) | 1 (1) |  |
| *Only to patients with chronic anemia, n (%)* | 34 (4) | 19 (4) | 5 (4) | 3 (3) | 5 (6) | 2 (3) |  |
| *Only if on EPO before ICU admission, n (%)* | 101 (12) | 56 (12) | 22 (16) | 8 (9) | 10 (11) | 5 (7) |  |
| *No, n (%)* | 690 (79) | 388 (80) | 104 (74) | 72 (83) | 67 (77) | 59 (87) |  |
| *No answer, n (%)* | 22 (3) | 13 (3) | 5 (4) | 2 (2) | 1 (1) | 1 (1) |  |

Hb = hemoglobin; RBCT = red blood cells transfusion; CAD = coronary artery disease; DCI = delayed cerebral ischemia; SvO_2_ = mixed venous oxygen saturation; PbtO_2_ = brain partial oxygen pressure; GCS = Glasgow Coma Score; ICHT = intracranial hypertension (>20 mmHg); CBF = cerebral blood flow; CO = cardiac output; DO_2_ = oxygen delivery; TRALI = transfusion-related acute lung injury; ABI = acute brain injury; EPO = erythropoietin

* Seldom (<25%); Sometimes (25-50%); Frequently (51-75%); Often (>75%)

**Additional file 2: Table S2.** Multivariable logistic regression analyses assessing associations between respondent characteristics and various transfusion policies for patients with acute brain injury (ABI). Data are reported as odds ratio (OR) and 95% confidence intervals (CIs).

|  | **QUESTION 1** | | **QUESTION 2** | | **QUESTION 3** | | **QUESTION 4** | | **QUESTION 5** | |
| --- | --- | --- | --- | --- | --- | --- | --- | --- | --- | --- |
|  | **OR (95% CIs)** | **p value** | **OR (95% CIs)** | **p value** | **OR (95% CIs)** | **p value** | **OR (95% CIs)** | **p value** | **OR (95% CIs)** | **p value** |
| **Continent (vs. Europe)** |  |  |  |  |  |  |  |  |  |  |
| *North America* | 1.84 [0.89-3.11] | 0.09 | 0.92 [0.38-2.24] | 0.86 | 0.64 [0.38-1.07] | 0.09 | 0.73 [0.41-1.31] | 0.30 | 0.75 [0.41-1.40] | 0.56 |
| *Central and South America* | 1.10 [0.22-5.42] | 0.91 | 1.04 [0.49-2.18] | 0.91 | 0.81 [0.16-4.42] | 0.81 | 0.89 [0.13-12.11] | 0.91 | 1.18 [0.66-2.08] | 0.21 |
| *Asia/Africa* | 1.75 [1.04-2.97] | 0.04 | 1.34 [0.12-14.34] | 0.81 | 0.82 [0.49-1.38] | 0.45 | 1.43 [0.82-2.49] | 0.20 | 3.08 [0.51-18.32] | 0.84 |
| *Oceania* | 2.06 [1.14-3.71] | 0.02 | 0.86 [0.40-1.83] | 0.69 | 0.71 [0.41-1.24] | 0.24 | 0.61 [0.31-1.19] | 0.15 | 1.27 [0.67-2.33] | 0.44 |
|  |  |  |  |  |  |  |  |  |  |  |
| **Certificate (vs. None)** |  |  |  |  |  |  |  |  |  |  |
| *Both* | 0.76 [0.45-1.23] | 0.31 | 0.82 [0.35-1.90] | 0.65 | 1.48 [0.88-2.49] | 0.13 | 0.72 [0.39-1.31] | 0.29 | 1.27 [0.71-2.25] | 0.41 |
| *Critical Care* | 0.99 [0.66-1.51] | 0.99 | 0.84 [0.42-1.65] | 0.61 | 1.19 [0.78-1.80] | 0.41 | 1.09 [0.69-1.74] | 0.69 | 0.90 [0.57-1.43] | 0.67 |
| *Neurocritical Care* | 0.59 [0.27-1.31] | 0.19 | 0.33 [0.10-1.11] | 0.07 | 1.11 [0.50-2.45] | 0.79 | 0.60 [0.24-1.46] | 0.26 | 0.85 [0.35-2.04] | 0.72 |
|  |  |  |  |  |  |  |  |  |  |  |
| **Specialty (vs. Intensive Care)** |  |  |  |  |  |  |  |  |  |  |
| *Anesthesiology* | 0.61 [0.42-0.89] | 0.01 | 1.46 [0.80-2.67] | 0.21 | 0.94 [0.65-1.37] | 0.77 | 0.83 [0.55-1.27] | 0.41 | 1.46 [0.96-2.22] | 0.07 |
| *Internal Medicine* | 0.99 [0.49-2.06] | 0.99 | 1.71 [0.54-5.38] | 0.36 | 0.49 [0.23-1.03] | 0.06 | 0.94 [0.41-2.11] | 0.88 | 0.70 [0.32-1.53] | 0.37 |
| *Neurology* | 0.76 [0.35-1.65] | 0.48 | 4.11 [1.07-15.75] | 0.04 | 0.87 [0.39-1.91] | 0.73 | 1.45 [0.61-3.45] | 0.40 | 1.53 [0.63-3.67] | 0.34 |
| *Pediatrics* | 2.01 [0.40-10.12] | 0.39 | 1.81 [0.13-24.31] | 0.65 | 0.59 [0.12-2.81] | 0.50 | 0.33 [0.02-4.13] | 0.39 | 0.26 [0.04-1.57] | 0.14 |
| *Surgery* | 1.07 [0.41-2.79] | 0.88 | 1.01 [0.19-5.15] | 0.99 | 0.87 [0.32-2.31] | 0.78 | 1.12 [0.41-3.33] | 0.77 | 0.84 [2.93-2.45] | 0.76 |
|  |  |  |  |  |  |  |  |  |  |  |
| **Years in the ICU (vs. < 5)** |  |  |  |  |  |  |  |  |  |  |
| *5-10* | 1.18 [0.75-1.87] | 0.46 | 0.77 [0.39-1.51] | 0.46 | 1.09 [0.69-1.71] | 0.70 | 1.19 [0.70-2.02] | 0.51 | 1.31 [0.79-2.16] | 0.29 |
| *11-15* | 1.37 [0.89-2.10] | 0.14 | 1.30 [0.66-2.55] | 0.44 | 1.09 [0.71-1.67] | 0.68 | 1.45 [0.89-2.38] | 0.13 | 1.35 [0.84-2.16] | 0.20 |
| *16-20* | 1.06 [0.59-1.89] | 0.82 | 1.18 [0.48-2.91] | 0.72 | 1.14 [0.64-2.03] | 0.64 | 1.18 [0.61-2.26] | 0.61 | 0.80 [0.43-1.48] | 0.48 |
| *21-25* | 1.11 [0.67-1.89] | 0.66 | 1.65 [0.69-3.96] | 0.26 | 1.06 [0.64-1.76] | 0.80 | 1.20 [0.67-2.19] | 0.53 | 1.19 [0.69-2.06] | 0.52 |
| *> 25* | 1.08 [0.62-1.90] | 0.76 | 1.55 [0.59-4.03] | 0.37 | 1.32 [0.76-2.31] | 0.32 | 2.32 [1.22-4.41] | 0.01 | 1.40 [0.76-2.57] | 0.27 |
|  |  |  |  |  |  |  |  |  |  |  |
| **Type of ICU (vs. Mixed ICU)** |  |  |  |  |  |  |  |  |  |  |
| *Medical ICU* | 1.20 [0.62-2.35] | 0.58 | 0.29 [0.13-0.64] | 0.02 | 1.33 [0.68-2.57] | 0.39 | 1.09 [0.52-2.26] | 0.81 | 1.17 [0.55-2.47] | 0.67 |
| *Neuro-ICU* | 0.97 [0.60-1.55] | 0.89 | 1.09 [0.52-2.27] | 0.81 | 0.93 [0.58-1.50] | 0.79 | 1.03 [0.61-1.77] | 0.89 | 1.22 [0.72-2.06] | 0.45 |
| *Pediatric ICU* | 0.78 [0.27-2.26] | 0.64 | 0.84 [0.17-4.08] | 0.83 | 0.98 [0.34-2.82] | 0.97 | 0.61 [0.15-2.37] | 0.47 | 1.62 [0.48-5.49] | 0.43 |
| *Surgical ICU* | 0.88 [0.52-1.49] | 0.63 | 2.36 [0.80-7.01] | 0.12 | 1.09 [0.64-1.86] | 0.73 | 0.81 [0.45-1.49] | 0.51 | 0.78 [0.44-1.37] | 0.39 |
|  |  |  |  |  |  |  |  |  |  |  |
| **Responsible for ICU (vs. Intensivist)** |  |  |  |  |  |  |  |  |  |  |
| *Anesthesiologist* | 1.89 [1.01-3.35] | 0.04 | 0.55 [0.23-1.33] | 0.18 | 1.30 [0.72-2.35] | 0.37 | 0.98 [0.50-1.91] | 0.94 | 0.91 [0.47-1.77] | 0.80 |
| *Mixed Responsability* | 0.92 [0.66-1.28] | 0.62 | 0.98 [0.57-1.67] | 0.94 | 1.18 [0.85-1.65] | 0.31 | 0.98 [0.67-1.43] | 0.91 | 0.98 [0.67-1.41] | 0.91 |
| *Neurologist* | 2.31 [0.64-8.35] | 0.19 | 0.68 [0.12-3.89] | 0.67 | 0.94 [0.29-3.08] | 0.92 | 0.92 [0.23-3.62] | 0.90 | 0.55 [0.16-1.89] | 0.34 |
| *Neurosurgeon* | 0.79 [0.34-1.81] | 0.57 | 4.36 [0.52-36.75] | 0.18 | 4.55 [1.70-12.02] | 0.02 | 3.02 [1.33-7.70] | 0.01 | 0.71 [0.28-1.79] | 0.47 |
|  |  |  |  |  |  |  |  |  |  |  |
| **Hospital size (vs. < 500 beds)** |  |  |  |  |  |  |  |  |  |  |
| *500-750* | 0.96 [0.66-1.41] | 0.85 | 1.52 [0.75-3.07] | 0.24 | 1.16 [0.81-1.68] | 0.16 | 1.12 [0.74-1.69] | 0.58 | 0.97 [0.64-1.45] | 0.87 |
| *750-1000* | 1.05 [0.69-1.61] | 0.79 | 1.92 [1.04-4.48] | 0.02 | 1.34 [0.88-2.04] | 0.41 | 1.18 [0.74-1.89] | 0.48 | 1.01 [0.63-1.59] | 0.98 |
| *> 1000* | 1.13 [0.71-1.79] | 0.59 | 2.24 [1.12-4.46] | 0.03 | 1.37 [0.88-2.04] | 0.45 | 0.62 [0.35-1.08] | 0.09 | 0.91 [0.55-1.51] | 0.73 |
|  |  |  |  |  |  |  |  |  |  |  |
| **Institution (vs. Academic)** |  |  |  |  |  |  |  |  |  |  |
| *Non-academic* | 1.15 [0.82-1.61] | 0.40 | 0.88 [0.53-1.46] | 0.63 | 0.98 [0.70-1.35] | 0.89 | 1.14 [0.78-1.65] | 0.48 | 1.06 [0.74-1.52] | 0.73 |
|  |  |  |  |  |  |  |  |  |  |  |
| **Triggers to initiate transfusions** |  |  |  |  |  |  |  |  |  |  |
| *Non-cerebral* | - | - | - | - | - | - | 0.20 [0.12-0.32] | <0.001 | 1.02 [0.59-1.74] | 0.93 |
| *Cerebral* | - | - | - | - | - | - | 0.81 [0.58-1.12] | 1.99 | 0.43 [0.31-0.60] | <0.001 |

In red, the p values < 0.05.

The five questions analysed are:

1. Which are the factors associated with a hemoglobin threshold of 7-8 g/dL to initiate RBC transfusion after acute brain injury?
2. Which are the factors associated with the use of “non-cerebral” triggers to initiate RBC transfusion after acute brain injury?
3. Which are the factors associated with the use of “cerebral” triggers to initiate RBC transfusion after acute brain injury?
4. Which are the factors associated with a hemoglobin threshold of 7-8 g/dL to initiate RBC transfusion after acute brain injury in the presence of any of the “triggers”?
5. In case of a randomized clinical trial, which are the factors associated with the comparison of a restrictive versus a liberal transfusion policy rather than a restrictive policy versus monitoring-guided?

The Hosmer and Lemeshow Test for the models of each question are 0.42, 0.28, 0.59, 0.37 and 0.69, respectively

**Additional file 2: Figure S1:** Regional distribution of respondents across continents.

**
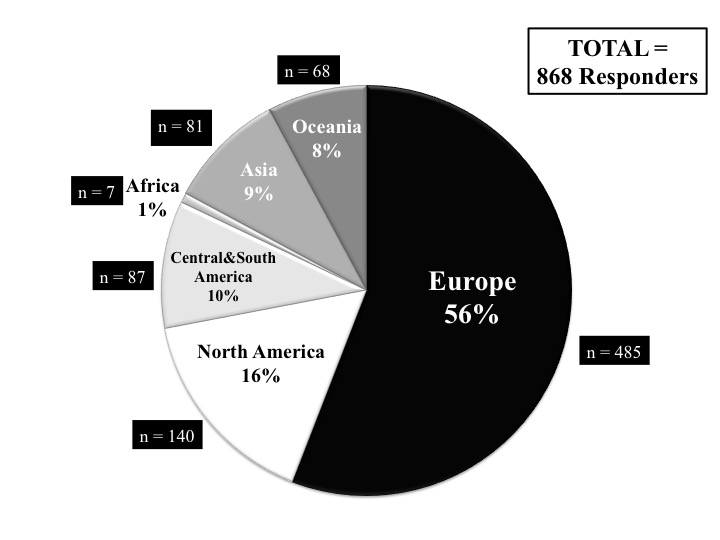
**

**Additional file 2: Figure S2:** Median threshold of hemoglobin (Hb) recommended to initiate blood transfusion in patients with acute brain injury in different geographic regions. a = p<0.05 vs. Europe; d = p<0.05 vs. Asia/Africa. Data are presented as median, IQR [IQR1/IQR3) and max values.


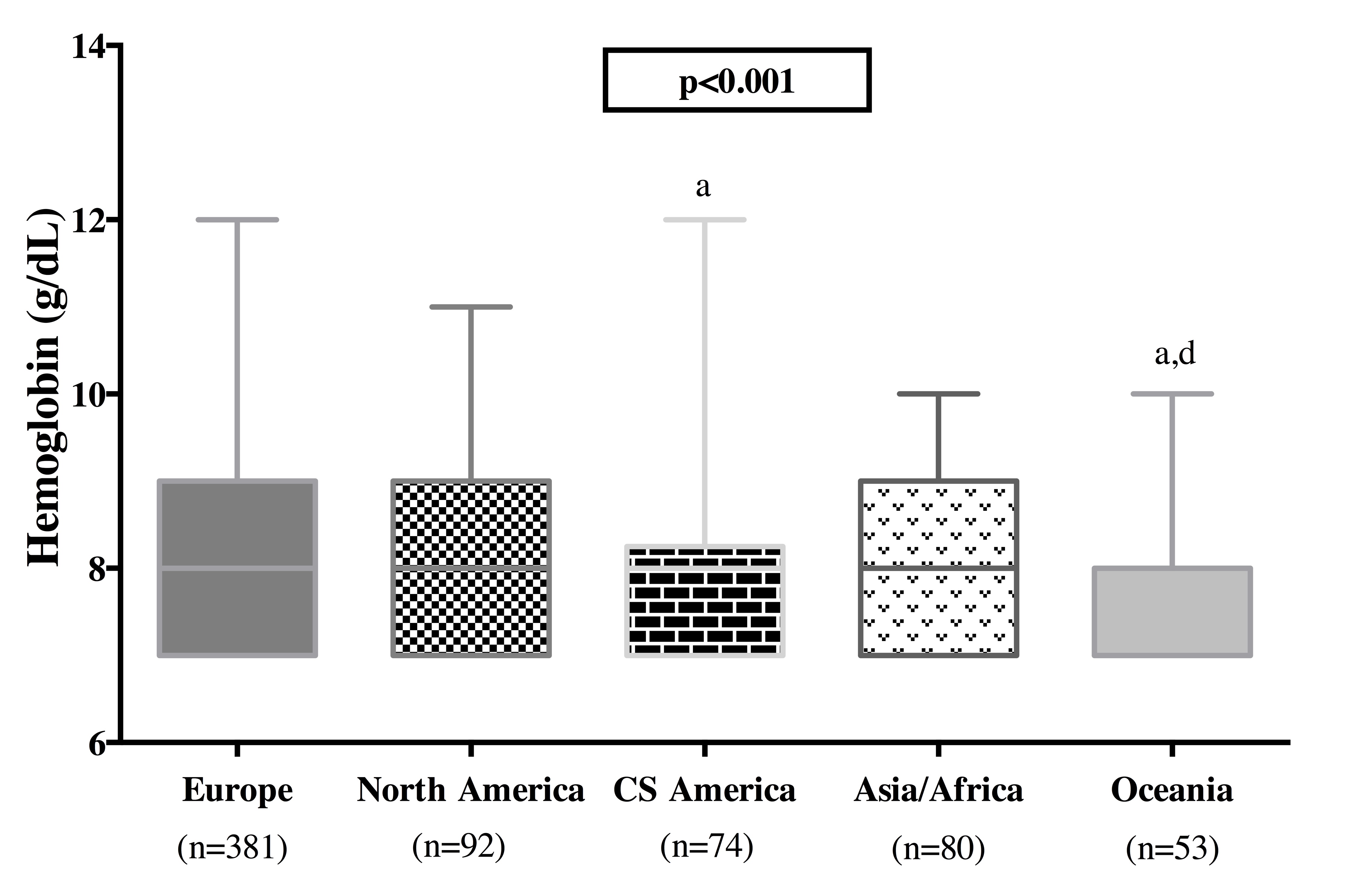


**Additional file 2: Figure S3:** Number of respondents reporting that different trigger factors (n) would influence their decision to initiate blood transfusion in patients with acute brain injury; black bars = “non-cerebral”; gray bars = “cerebral”.


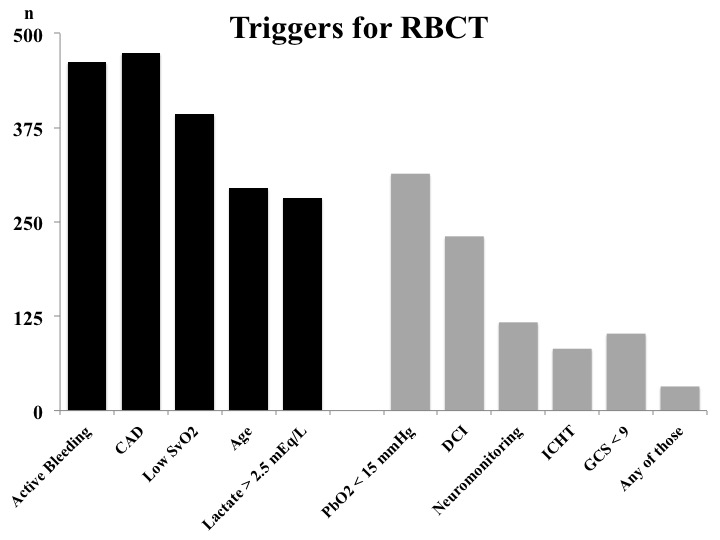


**Additional file 2: Figure S4:** Relative proportions of “non-cerebral” and “cerebral” trigger factors (%) used to initiate blood transfusion in patients with acute brain injury, according to geographic region


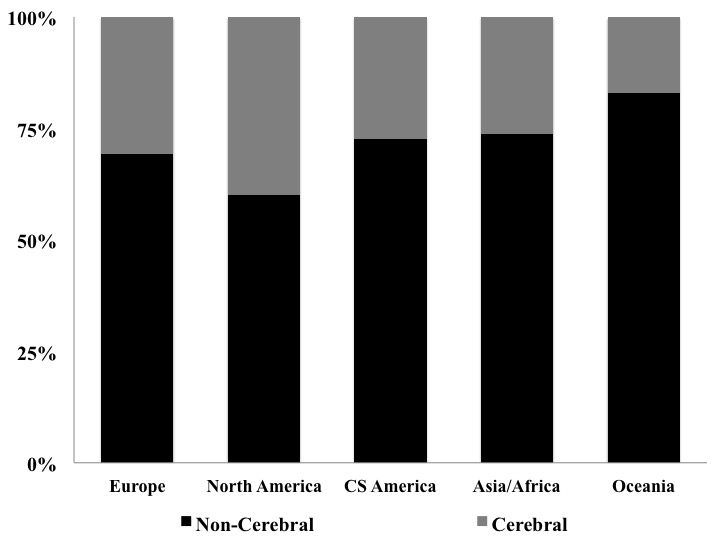


**Additional file 2: Figure S5:** Number of respondents (n) stating they would use a new threshold of hemoglobin (Hb) to initiate blood transfusion in patients with acute brain injury in the presence of a trigger factor.


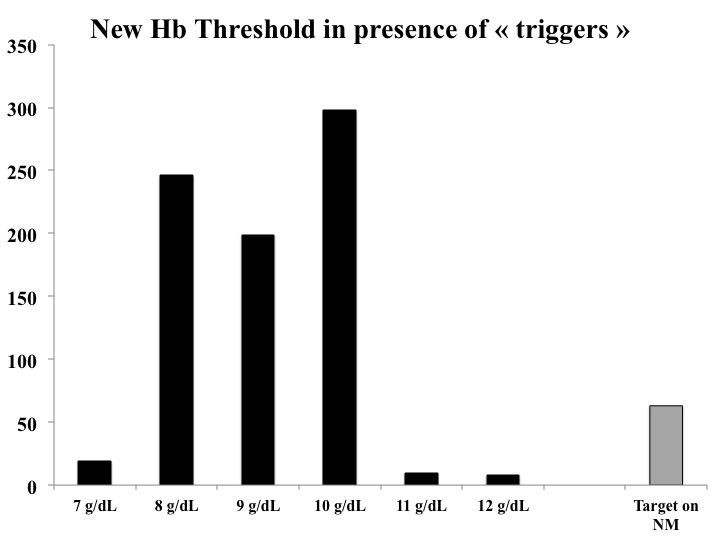


**Additional file 2: Figure S6:** Number of respondents (n) agreeing with the need for a randomized clinical trial (RCT) in anemic patients with from acute brain injury.


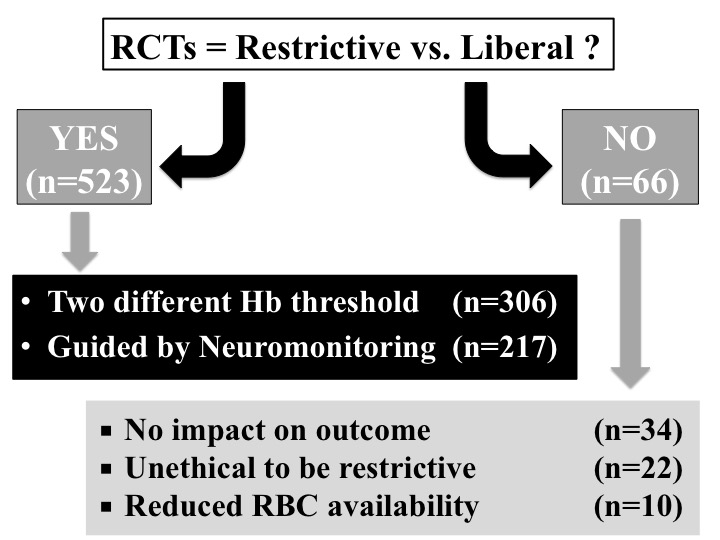

Supplement: Supplementary file 2 — Table S1. Transfusion policies among respondents. Table S2. Multivariable logistic regression analyses assessing associations between respondent characteristics and various transfusion policies for patients with acute brain injury (ABI). Figure S1. Regional distribution of respondents across continents. Figure S2. Median threshold of hemoglobin (Hb) recommended to initiate blood transfusion in patients with ABI in different geographic regions. Figure S3. Number of respondents reporting that different trigger factors (n) would influence their decision to initiate blood transfusion in patients with ABI. Figure S4. Relative proportions of noncerebral and cerebral trigger factors (%) used to initiate blood transfusion in patients with ABI, according to geographic region. Figure S5. Number of respondents (n) stating they would use a new threshold of hemoglobin (Hb) to initiate blood transfusion in patients with ABI in the presence of a trigger factor. Figure S6. Number of respondents (n) agreeing with the need for a randomized clinical trial (RCT) in patients with anemia from patients with ABI. (DOCX 884 kb) [file 13054_2017_1748_MOESM2_ESM.docx]
